# Exploring health-seeking behavior among adolescent mothers during the Ebola epidemic in Western rural district of Freetown, Sierra Leone

**DOI:** 10.1186/s12884-020-03521-7

**Published:** 2021-01-07

**Authors:** Hamida Massaquoi, Catherine Atuhaire, Gorgeous Sarah Chinkonono, Betty Nyawira Christensen, Hannah Bradby, Samuel Nambile Cumber

**Affiliations:** 1 Department of Women’s and Children Health, International Maternal and Child Heath, Uppsala University, Uppsala, Sweden; 2 Faculty of Medicine, Department of Nursing, Mbarara University of Science and Technology, Mbarara, Uganda; 3 Section for Epidemiology and Social Medicine, Department of Public Health, Institute of Medicine (EPSO), The Sahlgrenska Academy at University of Gothenburg, Gothenburg, Sweden; 4 Department of Sociology, Uppsala University, Uppsala, Sweden; 5 Office of the Dean, Faculty of Health Sciences, University of the Free State, Bloemfontein, South Africa; 6 School of Health Systems and Public Health, Faculty of Health Sciences, University of Pretoria, Pretoria, South Africa; 7 Institute of Health and Care Sciences, The Sahlgrenska Academy at University of Gothenburg, Gothenburg, Sweden

**Keywords:** Maternal health, Health-seeking behaviour, Adolescent pregnancy, Ebola, Sierra Leone

## Abstract

**Background:**

From 2014 to 2016, the largest Ebola outbreak in history threatened Sierra Leone and its neighbouring countries, Guinea and Liberia. The Ebola outbreak impacted pregnant adolescent girl’s access to prenatal care during the pandemic. The aim of this study is to understand health-seeking behaviour among adolescent mothers who were pregnant during the Ebola epidemic in Waterloo, Sierra Leone.

**Methods:**

The present qualitative study uses the “Three Delay” model, as a theoretical framework to understand and explore adolescent mother’s health-seeking behaviour through four focus group discussions with five participants in each discussion group. The data were analysed using thematic analysis.

**Results:**

A multitude of challenges were identified following the Ebola epidemic. The fear of contracting Ebola was a common reason for not seeking care or utilising services. This notion was perpetuated by perceptions in the community and participants personal experiences. Quarantines, national lockdowns, roadblocks, loss of income and extreme poverty were also identified as barriers to accessing health facilities during Ebola. The different encounters with health workers and the challenges that arose at the health facilities were subsequently additional discouraging factors influencing participant’s decision not to seek health care.

**Conclusion:**

Many of the pre-existing maternal health, societal and social-economic challenges were exacerbated during the Ebola. The epidemic also contributed new challenges such as public fear, mistrust towards health professionals and the health system. Greater emphasis needs to be placed on improving maternal care in general, but also improving preparedness for maternal care in case of future outbreaks, especially for the most vulnerable groups such as adolescent mothers.

**Supplementary Information:**

The online version contains supplementary material available at 10.1186/s12884-020-03521-7.

## Background

The West African Ebola epidemic (2014–2016) was the largest and most complex Ebola outbreak since the virus was first discovered in 1976 [1]. It is estimated that more than 1 million women in the affected regions of Guinea, Sierra Leone, and Liberia were pregnant during the outbreak [2]. These women were faced with a triple burden of mortality, by being at risk of dying from the Ebola virus disease (EVD), dying during pregnancy or childbirth [3]. Sierra Leone has one of the highest rates of maternal mortality in the world, with a staggering 1360 deaths per 100,000 live births in 2015 [4]. According to national statistics, 34% of all pregnancies in the country are related to adolescent pregnancy and 40% of maternal mortality occurs as a result of adolescent pregnancy [5]. The high rates of pregnancy may impair their future social, economic and political empowerment because many adolescent girls get pregnant before they complete primary level education [6]. Additionally, infants born to young mothers under the age of 20 also have a 50% higher risk of newborn mortality [7]. Findings suggest, that the level of maternal and newborn mortality had increased by 30 and 24% between 2014 and 2015 [8].

In light of the high rates of maternal and child mortality, the government launched the Free Health Care Initiative (FHCI) in 2010, providing free maternal and reproductive health for lactating mothers and children under five to utilise medical facilities [9]. The initiative led to a subsequent 45% increase in institutional childbirths [10]. However, mass relocation of health funds was utilised to fight the EVD, deprioritising obstetrical services and placing pregnant women at an increased risk of undetected complications and maternal mortality [2, 3, 11]. The risk of infection during childbirth, due to blood and fluid exposure resulted in health care workers refusing to treat pregnant women for fear of contamination [12]. During the EVD one-third of healthcare workers who died between April to September 2014, where maternal health care professionals. The EVD also affected the health-seeking behaviour and utilisation of health care services among pregnant women resulting in 11% decrease in deliveries at health facilities, 18% decrease in accessing antenatal care and 22% decrease in accessing postnatal care [8]. According to UNFPA report, pregnant adolescents are less likely to seek medical assistance because they have easier access to TBAs and community health workers [13].

In 2013, Sierra Leone was ranked among the 10 countries globally with the highest rates of adolescent pregnancy, with 28% of girls aged 15–19 years being pregnant or already experienced childbearing and about 40% of women aged 20–24 had already experienced childbearing before turning 18 [11, 14]. Due to the high rates of adolescent pregnancy, the government launched the nationwide programme of action in 2013 *Let girls be girls, not mothers! National strategy for the reduction of teenage pregnancy in Sierra Leone* (2013–2015). Unfortunately, the initiative was disrupted by the Ebola epidemic [5]. Additionally, emergency measures such as enforced quarantines households on affected by Ebola, three-days national lockdowns and school lockdown between June 2014–April 2015 were implemented as a strategy to reduce the spread of Ebola [6, 12].

Prior to the EVD, other factors such as cultural norms, beliefs about disease and perceptions of the quality of care provided, household power relations and social networks dictated health-seeking behaviour [3]. Furthermore, a multitude of challenges were identified following the Ebola epidemic, ranging from the economic recovery to (re) building trust in the health system to repurposing the Ebola Treatment Centres. One of the challenges not immediately apparent to many outside the country was the subsequent increase in adolescent pregnancy during the epidemic [3]. In April 2015, the Sierra Leonean government took controversial measures by banning visibly pregnant adolescents from finishing their education [6]. This policy was revoked in 2016, after international donors aided the government in supporting more than 14,500 pregnant girls, by initiating educational programmes and Community Learning Centres [4].

During infectious diseases outbreaks, sex, gender, and age play important roles, particularly for pregnant women who are more vulnerable to the effects of the disease [3, 6]. In Sierra Leone, adolescent girls were more susceptible to sexual exploitation, sexual assault and rape. Reports found that girls suffered far more violence and sexual exploitation when they were isolated, quarantined or moved to other areas to escape the EVD [3, 15]. Research from the Eastern region of Sierra Leone found that adolescent pregnancy increased by up to 65% in some target communities due to the socio-economic conditions affected by the EVD [16]. Limited research has been done pertaining to adolescent mother’s health-seeking behaviour during Ebola. The study aimed to explore health-seeking behaviour and the use of health services among adolescent mothers who were pregnant during the Ebola outbreak in Freetown, Sierra Leone.

## Methods

### Study setting

Sierra Leone has 4 provinces and 14 districts. The Western Area province is divided into an urban and rural district. The study was conducted in Waterloo, which is the capital in the Western Rural Area. The Western Rural Area has a population of 444,270, with (48.1%) of the population living in the Waterloo. The ethnic composition is predominantly Temne (48.7%) followed by a large minority of Mende (12.8%) and Limba (11.3%); and the majority of the population are Muslim (72%). Waterloo is one of five chiefdoms in the rural district [17].

The District Health Management Team (DHMT) has a total of 317 registered staff medical and non-medical staff working in health facilities in the District. The facilities available in Western Rural Area: 12 Community Health Centres (CHC), 20 Community Health Posts (CHP), 21 Maternal Child Health Post s (MCHP) and 1 hospital. Traditional medicine is also considered a part of the primary health care system in Sierra Leone. Western Rural Area reported the last two EVD cases on 20 April 2015. During the EVD, this area was considered an epicentre of the outbreak. The cumulative number of confirmed cases is 1164 for the area [18].

### Study design and population

This study was a descriptive study design employing qualitative methods of data collection to explore attitudes and perceptions from adolescent mothers. This data was obtained in June 2016 and was guided by the framework of the “Three Delays” model to understand maternal outcome and how the EDV has influenced health-seeking behaviour and the utilisation of health services according to each delay: (1) deciding to seek appropriate medical help for an obstetric emergency; (2) reaching an appropriate obstetric facility; and (3) receiving adequate care when a facility is reached [19].

This study targeted adolescent mothers aged 15 to 24 who have been pregnant during the EVD outbreak from September 2014 to April 2015 and had been in contact with maternal health care services during their pregnancy or after childbirth.

### Sample size estimation

All participants were recruited through homogeneous sampling. With assistance from the local NGO coordinators in Waterloo who helped identify eligible participants. Twenty adolescent mothers were approached and recruited as participants. In a study with a relatively homogeneous population, using a semi-structured guide three Focus Group Discussions (FGDs) will likely capture 80% of the most broadly shared themes and topics [20]. The point of saturation was reached after 4 FGDs.

### Data collection

Four FGDs with five participants in each group were employed as a primary mode of data collection to understand social norms, expectations, and experience and how community members form perceptions and attitudes to influence behaviour. The FGDs also aimed to produce group interactions and stimulate discussions based on shared experiences, realities, attitudes and perceptions toward health-seeking behaviour [20]. The duration of the FGDs was approximately 40–60 min. The principal researcher moderated all of the FGDs in the local language Krio which is a mixture of English and indigenous- and afro-descent language. The discussions were conducted in a local school building in a private office. All participants were recruited through in collaboration with ‘Health Alert’, an NGO known for its routine involvement and work with vulnerable girls and young women in Urban and Rural Western District Area.

### Data collection tools

A focus group discussion guide was developed for the study to guide the conversations and provide prompts as attached in Additional file 1. Participants were also asked to comment on a short summary of the VSO report “*Exploring the impact of the Ebola outbreak on routine maternal health services in Sierra Leone”* [8]. The questions from the guide was also inspired by each delay in the Three Delay Model to help determine where improvements could best be made to reduce the risk of maternal mortality [19]. The guide was tested during a pilot semi-structured interview in order to help modify the questions. Data from the pilot interview was not included in the data set. The questions in the FGD guide included both broad open-ended questions regarding general perceptions on health care and specific questions regarding challenges relating to health care access. All the FGDs were audio-recorded and notes were taken during the FGDs to supplement the audio recording.

### Data analysis

The FGDs were transcribed verbatim, and translated from Krio to English by the first author. The transcripts were analysed in depth after re-reading the transcripts. Thematic analysis, as described by Braun and Clarke was found to be an appropriate framework for analysis due to the explorative nature of the study, the emergent design of the data collection, and the aim and objectives of the study [21]. Codes and themes were found manually by highlighting the transcript in sections and identifying the codes generated through the FGDs. The data were then rearranged according to the identified patterns. Furthermore, codes were then combined with more abstract sub-themes and themes. An inductive approach was used to find the correlation between codes, sub-theme, theme and the framework. Lastly, the dataset was re-read to ensure that themes correlated to the full dataset and that important data had not been missed.

## Results

**Table:** 

**Respondents’ characteristics**	**FGD (description and number)**
Total participants	20
Age range	15–23 years
Marital status	married [2]
	unmarried [18]
Educational status	completed primary level [17]
	no education (3)
Place of delivery	health facility [5]
	traditional birth attendant [15]
Contact with health facility	only during pregnancy [6]
	only post pregnancy [11]
	both during and after pregnancy [3]
Personal loss due to Ebola	loss of relative or [5]

The data were grouped under categories within the three delays framework. The main themes reflect a combination of ‘The Three Delay Model’ and the key subjects that were discussed in relation to each delay. The first theme underlines how fear on a community and individual level influenced participant’s decision to not seek care. The second theme highlights how an enforced measure such as quarantine led to financial strain which became an additional barrier to health care access during the outbreak. The third theme describes the different encounters participants had with health care workers at the health facility and how this negatively affected their behaviour towards health-seeking. Illustrative quotes, aimed at representing the main findings of the theme are presented in italics.

### First delay: fear of EBOLA

#### Fear in the community

Some participants had stopped seeking health care at hospitals and clinics after giving birth, due to fear. The adolescent mothers discussed how the fear of Ebola in the community was a major challenge, which caused a general sense of discouragement toward health-seeking. According to the adolescent mothers, the reactions to the outbreak, the circulating rumors and misconceptions about Ebola were most prominent at the beginning of the outbreak, but speculations reduced as the number of Ebola victims started declining. Participants were confronted daily with news of the outbreak and unfortunate experiences of loss in their communities. Some participants had been skeptical of the EVD describing how the fast spread in their communities awoke superstition and conspiracy theories, which shaped their perceptions toward health care facilities. When participants were asked what community, members said that discouraged them from health-seeking, they supported each other’s comments by saying:

*FGD3, P3:“They encouraged us to give birth at home. Because if you go to the hospital you won’t come out alive.”*

*FGD3, P1:“They will leave you there (in the hospital) until you die.”*

*FGD3, P4:“If they come and take you with the ambulance and you don’t return then they (members in the community) will believe it is an Ebola case.”*

*FGD3, P5:“Some areas always had bad rumors, if you go to the hospital for two or three days the community will say Ebola took you away.”*

The fear of contracting Ebola led to many of the participants preferring home-births by traditional birth attendants (TBA), in spite of many TBAs refusing to provide care during the epidemic, due to the lack of protective equipment and fear of contracting Ebola. Home delivery was said to be encouraged by people in the community, due to preconceived notions and ignorance about the transmission of the EVD. The decision to seek health care or deliver at home was described as a terrifying dilemma many participants faced during their pregnancy. Death was viewed as the ultimate outcome which evoked feelings of distress and hopelessness.

Some of the mothers who had a home-delivery said they had not sought care after their delivery because they were scared, while others participants had avoided going to the hospital for a whole year, due to the fear of contracting Ebola.

*FGD3, P3:“People were scared, even if they don’t get the sickness but they will die of fear because they refuse to go to the hospital. If you feel pain you’ll be scared that if you go to the hospital you will die. You will end up being so scared you deliver at home and then you over bleed, you die.”*

#### When personal loss leads to fear

The experience of personal loss was another barrier to seeking health care. The loss of friends, close and distant relatives was described as discouraging, scary and traumatizing experiences, that not only led participants to grief but also increased the fear of losing their own lives. Some participants explained how they were separated from relatives due to loss, while others moved in with extended family outside affected areas out of fear of contamination.

*FGD2, P3: “Like me, my sister she had the same problem. When we went to the hospital with her they would not give her treatment and then she lost her life, so we were all scared of going to the hospital because she lost her life. Anyone who went there to visit her they would send them away. She was so sick that God took her life. So we were so scared of going to the hospital, this sickness that came scared us all (…)”*

Participants explained how their experience of personal loss made them live in fear. Witnessing the sudden death of a family member up close was justification for participants’ mistrust of the health system. The adolescent mothers described how it made them feel unsafe in their community, especially after witnessing Ebola ambulance entering their neighborhood and spraying chlorine and unknown liquids.

### Second delay: reaching facility

#### The financial burden

The lockdown had dire consequences for people with low socio-economic status who were already struggling to survive as indicated in the quote below:

*FGD4, P4: “The reason why I was suffering more was because of the lockdown, at that time my husband was a driver and cars were not allowed to go anywhere, and if he cannot get customers how should we get by.”*

During the three-day lockdown participants were said to have lost their income because they were cut off from earning a living and subsequently unable to access health care, food and water. Local and weekly markets were also banned, affecting participants with local businesses. The government also shut down small facilities, converting hospitals and clinics into Ebola treatment centres, furthering the distance to health facilities, subsequently worsening the conditions for pregnant women, who already faced limited access to adequate health care.

For some participants, transportation played a significant role in the perceived spread of the EVD. Therefore, the continuous and rapid spread of the EVD was of great concern for many of the adolescent mothers both during and after their pregnancy. As a result, some participants preferred walking or taking a motorbike instead of the local boda boda (mini-bus), which on one hand was considered to be a more affordable option, but on the other hand were usually more crowded. Local drivers also refused to take passengers from certain areas in Waterloo with increased spread of EVD or high death rates, which became a major constraint for some participants, forcing them to walk for miles before being able to access transportation.

### Third delay: experience at health care facilities

#### Health care workers’ behavior towards adolescent mothers

One of the main challenges participants identified was the unfriendly attitude among health care workers. Adolescent mothers were often met with unwelcoming and negative attitudes among health care workers. This behaviour discouraged the adolescent mothers from utilising health facilities, which led to them seeking aid from traditional birth attendance and private facilities or just staying at home when ill, as described in the quotes below:

*FGD2, P3:“Because we saw the type of treatment people received so even if you go for treatment you will be discouraged and go back, go sit at home, because you will see how they treat the next person.”*

*FGD1, P5:“When you go to the hospital the nurses, when you want to sit down they will yell at you, they will yell at you as if you are a mad person. When we go to the hospital they treat us badly during Ebola.”*

Moreover, a few participants suggested that health care workers’ behaviour had worsen during Ebola, due to their fear of contamination. The young women were frustrated with no longer being examined properly. Nurses no longer touched pregnant women without gloves or examined or auscultated the fetal heart in order to identify the fetus positioning or gestational age. It was reported that fear among nurses and midwives were strong, because not all facilities had received training, and some did not have access to protective equipment such as gloves and masks at the beginning of the epidemic:

*FGD1, P5:“So when I went there they said that right now they do not touch people, so I should go and sit down for a while, so I decided to return home. When I came home, I did not go to the hospital again, because I have tried not once but twice and they do not want to touch me, so I do not want to go”*

All the FGDs had reports of health care workers verbally insulting and stigmatising the adolescent mothers because of their age. The participants, nurses’ viewed adolescent pregnancy as an intentional act that the girls chose instead of completing their education. It was indicated that this behaviour and perception existed before the outbreak, but it exacerbated due to the increased level of adolescent pregnancy.

*FGD2, P5:“(….) The first thing she said (referring to the nurse),“you children now a days when the put you to school what do you come with, what is the profit you bring to your parents, only pregnancy” (…)”*

#### Financial barriers to health

Participants were aware of the Free Health care initiative the government had implemented for children under 5 years of age and lactating mothers, but according to some of the adolescent mothers, the initiative was affected by the reallocation of health care funds, prioritising health services for the EVD. Therefore, health care workers were still charging patients for health care expenses. Due to their low socioeconomic background participants were unable to pay for the unexpected charges, which became a common reason for not seeking care during this time. One participant mentioned how health workers would try to sell drugs to them for their financial gain and many times the only alternative they had was to buy traditional medicine.

*FGD2, P2:“Then they prescribe drugs for you to go and buy and as soon as you leave the hospital they call you back “come we have drugs for sale, come we will sell it to you ”(...) They deal with money at the hospital right now.”(…)* ***“****they do not give you any drug that is the thing, we have to take the children to the pharmacy to provide drugs for them the country way (traditional medicine) wash the child with country medicine.”*

Socio-economic inequalities were also discussed and several participants described how they were left largely unattended because they failed to pay the required fee and how partiality was shown to those who had the means to provide the charged fee. Paying out-of-pocket costs for medical expenses was reported as a common phenomenon, especially during Ebola. At some health facilities nurses required patients to pay a fee whenever they visited, demanding that they “greet the table”, which was an expression commonly used when asking patients to pay user fees before being attended to;

*FGD2, P1:“When you go to the hospital they will tell you to greet the table (...) They say “if you do not greet the table we won’t attend to your child”. So if you don’t have that money it does not matter how long you sit there they won’t attend to you. You just have to return home in peace. So that is why if you do not have money you don’t go to the hospital, you will sit at home.”*

Participants described how health care workers were hesitant to expose themselves to potentially infectious bodily fluids and therefore they were more kind to patients if they could document a negative Ebola test before receiving treatment. At the beginning of the epidemic the test only cost 17,000 Leones equivalent to 2.35 dollars, which was considered very expensive for those living below the poverty line. The cost for the Ebola screening gradually increased during the outbreak and some participants ended up paying 70,000 Leones, equivalent to 10 dollars.

*FGD3, P2:“If you do the test (Ebola test) and you have done everything they will speak to you nicely. As long as you have done the test and you can show documentation saying you have done the test. But the test is very expensive to take, sometimes they will say 70,000 Leones, and not all pregnant women have that kind of money.”*

*FGD3, P3:“If you have or if you don’t have money, and even if you say you don’t have money, they will tell you to go and find money “ go to the man who got you pregnant so he can pay the 17,000 Leones so you can do the test”. Some people cry because of the condition.”*

Women who did use the service before Ebola acknowledged that the general care they received was of better quality, because the queuing system was in order before with a “first go, first served” policy and one was almost guaranteed to be seen and cared for by health professionals. As expressed by participants, nurses and midwives were considered less compassionate as they feared for their own lives and irrespective of how sick the women were when arriving at the facility, they were not attended to unless they paid for service charges or could provide some evidence of their Ebola status.

## Discussion

The aim of the study was to explore how the EVD outbreak influenced the use of health services among adolescent mothers who were pregnant during the outbreak. This study, therefore, attempts to shed light on attitudes, perceptions, experiences and barriers participants faced in attempt to health-seeking during the EVD.

The results from this study contributed to the Three Delay Model by indicating how an external factor such as the EVD added new complexities to each of the delays. The Three Delay model also recognises the interrelated factors that create barriers to health-seeking. Although there were several factors affecting adolescent mother’s health-seeking behaviour the association between these factors were not always linear. Rather fear and socio-economic factors cut across the three delays. The fear of contracting the EVD from both participants, community members and health professionals was an underlying factor, in (delay 1 and 3). Some participants fear of taking public transportation (delay 2).

First, the scientific literature suggests, mistrust and fear were exacerbated in the community as conspiracy theories and rumours regarding the EVD and health facilities spread. Rumours and misconceptions also acted as a direct barrier to health-seeking in both Guinea and Liberia [22]. Jones et al. reported that as a result of fear participants would often arrive at the facility after conditions had worsened, leading to poorer health outcomes [8].

Secondly, many participants feared being exposed to EVD in health facilities which prevented them from seeking obstetric care. Instead, most of the participants decided to seek a TBA and deliver at home without qualified assistance translated to obstetric complications and fatal neonatal or maternal outcomes. Similarly, a Liberian study found that births in public facilities decreased from about 54 to 27% during EVD, because women were afraid of seeking health care in government hospitals. The study also found a decrease in supply as many health care workers did not want to treat pregnant women due to fear [23].

Thirdly, the loss of relatives and community members also added feelings of mistrust towards health care facilities as they failed to provide adequate care and treatment, which became another reason for not seeking care. Research suggests pregnant women trusted TBA more because they were less likely to discontinue maternal services due to fear [22, 23].

Fourthly, the lockdown and quarantine, lack of income and road restrictions were considered as significant barriers to accessing prenatal and obstetric care. In Guinea, resources and health facilities were also directed towards controlling the EVD, which limited the access to health facilities for pregnant adolescents [24]. This was also reflected in Liberia, where the largest group unable to access prenatal care during the EVD were pregnant women [25]. Evidence shows that ambulances were available for referrals prior to EVD, but the numbers of vehicles were limited and not always in working order. When ambulances were available, the poor infrastructure was another existing challenge for referring women [8]. Participants from high spread EVD areas in Waterloo usually had to walk for miles before they could access public transportation, as many drivers refused to take passengers from these areas. During the EVD motorbikes, even though more expensive, became the preferred mood of transportation in both rural and urban areas [13, 25].

The perceived discriminatory and disrespectful behaviour from midwives and nurses caused adolescent mothers to avoid seeking health care, combining elements in the third delay (the quality of care) with the first delay (previous experience with health care providers) [19]. Moreover, the study considered maternal age as a factor that could potentially become a barrier to health-seeking, considering that adolescent mothers statistically are proven to be at a higher risk of maternal mortality [5]. However, findings revealed that the quality of care was not only determent by participants’ personal assessment of service delivery; perceptions were also shaped and influenced by the experience and opinions of community members. Evidence reports that the perception of adolescent pregnancy out of wedlock in most sub-Saharan African settings are negative [3, 23, 26]. Single adolescent mothers are in most communities considered to be less respectable, a disgrace to their parents and they are deemed as idle and promiscuous, usually subjected to shame, gossip and rejection in their community [26]. These findings are aligned with a previous study from rural Sierra Leone, where adolescent mothers were more likely to be stigmatised and experience additional barriers [26].

The inability to correctly diagnose the EVD especially during the beginning of the epidemic was also an interesting finding. The fear of pregnancy symptoms being mistaken for Ebola symptoms caused participants to avoid seeking health care. The hidden fees and out-of-pocket payments participants encountered at the health facility was also a barrier to receiving quality healthcare. This barrier was also found in among the rural population in Sierra Leone during Ebola [26]. While some participants bribed their way through the system others sought to traditional medicine and other alternatives. Out-of-pocket expenses for unexpected charges raised concern among participants, causing confusion as to whether or not the Free Health Care Initiative was still available. In Liberia evidence revealed that pregnant women and women suffering from obstructive labour were also refused treatment from health care facilities during Ebola because they were unable to pay the required health charges, subsequently leading to some women dying from maternal mortality [3].

### Limitation

The study had several limitations. Firstly, the findings are not representative of other parts of Sierra Leone and are restricted to Western rural Area. The sample only consisted of adolescent mothers who had been in contact with prenatal and obstetric services. Due to the high rate of participants who delivered with assistance from a TBAs, it is possible that many adolescent mothers did not have any contact with health facilities throughout their pregnancy.

## Conclusion

The study found that the underlying reason for poor health-seeking behaviour among adolescent mothers was due to the fear of contracting the EVD and other barriers such as lack of access, excessive cost of medicines and poor treatment from healthcare workers. Because of the pre-existing frailties in the health systems many of the central public health concerns in the countries spiralled out of control during the epidemic as the rates of adolescent pregnancy, maternal mortality reached new peaks, exacerbating the health and socio-economic conditions for adolescent mothers who were already struggling to survive. The government in Sierra Leone is now left with a responsibility to take urgent action toward improving access to maternal health.

## Recommendation

Pre-Ebola initiatives such as the FHCI led to an increase in institutional childbirth. However, during the EVD certain pre and postnatal services were no longer free of charge. Therefore, additional attention must be paid to improving and strengthening policies to includes EVD and disease outbreaks in order to deal with fear and mistrust and some of the other underlying challenges that exacerbate due to the EVD [27, 28].

Moreover, policies should also ensure that adolescent mothers are allowed to continue their education to promote their future socioeconomic and political empowerment [4]. In accordance with articles 24 and 27 of the Convention on the Rights of the Child, States parties should provide health services that are sensitive to the particular needs and human rights of all adolescents [29]. Lastly, in order to improve adolescent’s uptake in Sierra Leone efforts must be made towards improving health systems and promoting initiatives that address the barriers related to the first, second and third delay. Doctors, nurses and midwives must be adequately trained for future responses to EVD or other disease outbreaks, enabling them to safely continue caring for patients. Moreover, developing sustainable measures such as training TBAs and equipping them with proper supplies is crucial in order to improve the capacity to manage the future spread of disease.

## Supplementary Information

**Additional file 1.** FGD guide, Health-seeking behavior.

## Data Availability

Interview scripts can be shared by the first author, Hamida Massaquoi upon request but restrictions apply under license for the current study. The data may be made publicly available upon reasonable request.

## Abbreviations

EVD: Ebola virus disease
FHCI: Free Health Care Initiative
DHMT: District Health Management Team
CHC: Community Health Centres
CHP: Community Health Posts
MCHP: Maternal Child Health Post
FGD: Focus Group Discussions
NGO: Non-Government Organization
TBA: Traditional Birth Attendant
P: Participant
UNFPA: United Nations Family Planning Association
HIV/AIDS: Human Immunodeficiency Virus / Acquired Immune Deficiency Syndrome
