# Supplementary material for: Exploring health-seeking behavior among adolescent mothers during the Ebola epidemic in Western rural district of Freetown, Sierra Leone
Source: BMC Pregnancy Childbirth. 2021 Jan 7;21:37. doi: 10.1186/s12884-020-03521-7 (PMC7791629; doi:10.1186/s12884-020-03521-7)
Supplement: Supplementary file 1 — Additional file 1. FGD guide, Health-seeking behavior. [file 12884_2020_3521_MOESM1_ESM.docx]

# Interview guide for focus group discussion

### Introduction

- Start by thanking participants for wanting to take part in this focus group discussion.
- Give a short introduction of myself

**Explain the purpose:**

The purpose of this focus group discussion is to understand your current thoughts and feelings about seeking healthcare during and after pregnancy. The information given during this discussion will be used as data collection for my thesis. Copies of informed consent and confidentiality forms will be provided for each participant and read aloud for the benefit of those who cannot read. Participants will be provided with an opportunity to ask questions.

**Explaining ground rules**

Inform participants that there are no right or wrong answers and that they should respect each other by, not interrupting or telling anyone about what we have discussed in the focus group. Emphasise the fact that honest opinions and different viewpoints are appreciated, even if they disagree with other participants.

**Explain the role of the moderator**

Guide the discussion and not actively participate, and thus encourage participants to interact with one another and respond to each other’s comments.

**Icebreaker**

Going around the circle and having each person introduce themselves (including members of the research team)

**Presentation of the focus/problem to be discussed:**

Introduce the VSO summary about health-seeking behaviour during the Ebola.

**Guiding questions**:

1. Ask participants to comment on the report: “What are your thoughts about this? “
2. What are your thoughts about the health care system in Sierra Leone?
3. What is your biggest challenge in regards to seeking health care as a teenage mother?
4. What are some of the positive/negative things about health care in Sierra Leone?
5. How was your access to health care during/after Ebola?

**Probes questions:**

1. “Would you explain further?”
2. “Would you give an example?”
3. “What do you think about what she said?”
4. “Has anyone experienced something similar?”

**Summary question:**

After the brief oral summary, the question asked is: "Is this an adequate summary?"

**Concluding question:**

Of all the things we’ve discussed today, what would you say are the most important issues you would like to express about seeking health care?
